# Effect of Depth of Total Intravenous General Anesthesia on Intraoperative Electrically Evoked Compound Action Potentials in Cochlear Implantation Surgery

**DOI:** 10.1155/2019/6838506

**Published:** 2019-12-01

**Authors:** Ala”a Alhowary, Abdelwahab Aleshawi, Obada Alali, Manal Kassab, Diab Bani Hani, Khaled EL-Radaideh, Firas Alzoubi

**Affiliations:** ^1^Department of Anesthesiology, Faculty of Medicine, Jordan University of Science and Technology, Irbid 21110, Jordan; ^2^Faculty of Medicine, Jordan University of Science and Technology, Irbid 21110, Jordan; ^3^Department of Maternal and Child Health, Faculty of Nursing, Jordan University of Science and Technology, Irbid 21110, Jordan; ^4^Division of Otolaryngology, Department of Special Surgery, Faculty of Medicine, Jordan University of Science and Technology, Irbid 22110, Jordan

## Abstract

**Purpose:**

This study aims to compare the effect of the depth of total intravenous anesthesia (TIVA) on intraoperative electrically evoked compound action potential (e-ECAP) thresholds in cochlear implant operations.

**Methods:**

Prospectively, a total of 39 patients aged between 1 and 48 years who were scheduled to undergo cochlear implantation surgeries were enrolled in this study. Every patient received both light and deep TIVA during the cochlear implant surgery. The e-ECAP thresholds were obtained during the light and deep TIVA.

**Results:**

After comparing the e-ECAP means for each electrode (lead) between the light and deep anesthesia, no significant differences were detected between the light and deep anesthesia.

**Conclusion:**

The depth of TIVA may have no significant influence on the e-ECAP thresholds as there was no statistical difference between the light and deep anesthesia.

## 1. Introduction

Cochlear implants are surgically implanted neuroprosthetic devices that supply a sense of sound for patients with moderate to profound sensorineural hearing loss. However, in order to obtain favorable speech understanding, long-term rehabilitation and immediate adjustment of stimulation objects are mandatory. Adjustment of the speech processor is a complicated process in pediatrics and requires an experienced audiologist [1].

Different electrophysiological objective measures can be used along with the subjective judgment, namely, intraoperative electrode impedance measurement, electrically evoked stapedial reflex, and the electrically evoked compound action potentials (e-ECAPs) [2, 3]. These measures can be used to objectively fit the sound processing system [4, 5]. At our center, we commonly use the e-ECAP thresholds. The e-ECAP recordings can be generated intraoperatively or postoperatively. When done intraoperatively, the child is still under general anesthesia. This allows the audiologist to apply high stimulation levels which result in a high success rate of recording accurate e-ECAP responses [6].

The knowledge of the effects of different anesthetic agents on the aforementioned different electrophysiological objective measures is important to optimize the outcome of pediatric cochlear implantation. The ideal anesthetic technique for cochlear implant surgery is the one that has no effect on the evoked auditory responses that were measured [3]. Several studies that have investigated the influence of anesthesia on electrically elicited stapedius reflex threshold (ESRT) measurements revealed that the total intravenous anesthesia (TIVA) gives more consistent responses than the volatile anesthetics [3, 4]. However, there are insufficient data in the literature concerning the effect of anesthesia on the e-ECAPs. Eftekharian et al. concluded in their study that the depth of general anesthesia (inhalational) can have significant influence on the e-ECAP thresholds, and it is important to reduce the depth of anesthesia to achieve better results [5]. Also, in a previous study, the authors compared the effect of the TIVA and the effect of inhalational anesthesia on the e-ECAPs, which resulted in higher e-ECAP thresholds within the inhalational anesthetic group [7].

Current cochlear implant systems consist of a stimulus receiver with a multichannel electrode array that are surgically implanted and an external sound or speech processing unit (usually worn behind the ear) that controls the implant over a transcutaneous radio frequency link [4, 8]. To the best of our knowledge, no studies have been performed to compare the effect of the depth of TIVA on the intraoperative e-ECAP thresholds. The aim of the present study is to compare the effect of the depth of TIVA on the intraoperative e-ECAP thresholds in cochlear implant operations.

## 2. Materials and Methods

After obtaining the formal Institutional Review Board (IRB) approval in our tertiary care university hospital, we prospectively identified and included those patients who were scheduled to undergo cochlear implantations surgery between January 2018 and April 2019. (This is the period of time when the referral system for cochlear implant was active. The referral system was ceased after that. Accordingly, efforts were made to allocate all cases.) A total of 39 patients aged between 1 and 48 years were enrolled in this study. All individuals were cochlear implant candidates diagnosed with bilateral, severe to profound congenital sensory neural hearing loss. Those patients with acquired type of hearing loss, auditory neuropathy, or inner ear malformations were not enrolled in the study. For all individuals, a written informed consent was obtained from the adult patients or one of the parents or the legal guardian for the children. All patients (or their parents) received detailed information about the study and gave the consent for participation.

### 2.1. Anesthetic Settings

After the preoperative assessment, all individuals had the American Society of Anesthesiologists' physical status of I to II. The patients were not premedicated. On arrival to the operating room, standard intraoperative monitors (pulse oximetry, noninvasive arterial blood pressure, and electrocardiogram) were applied and baseline values were recorded.

Induction of general anesthesia was carried out by nitrous oxide 70% administration along with oxygen 30% and sevoflurane 6–8% inspired dial concentration or with intravenous propofol 3 mg/kg depending on whether an intravenous line is secured before the induction. After the loss of consciousness, intravenous fentanyl 2 *μ*g/kg was administered followed by intravenous atracurium 0.5 mg/kg to facilitate tracheal intubation with an appropriately sized endotracheal tube.

A continuous infusion of fentanyl 0.3–0.6 *μ*g/kg/hour and propofol 4–8 mg/kg/hour was done intravenously in a titrated dose depending on the hemodynamic responses. The inhalational anesthetic agents (i.e., sevoflurane and nitrous oxide) were ceased after the induction.

Monitoring of the depth of anesthesia was conducted by the Bispectral Index (BIS) during the induction, maintenance, and measurement of the e-ECAP thresholds. The BIS was measured by a BIS monitor (BIS; Aspect Medical Systems, Newton, Massachusetts). The BIS monitor uses an electroencephalogram signal to measure the depth of anesthesia which then calculates the depth of anesthesia by the BIS as a number. A BIS less than 40 demonstrates very deep anesthesia, 40–60 means surgical anesthesia, 61–80 means light anesthesia, more than 81 demonstrates sedation, and 100 shows full awareness. All anesthetic interventions were carried out by a single consultant pediatric anesthesiologist.

### 2.2. Surgical Settings and Determination of the Auditory Thresholds

Cochlear implant surgery was performed by a single consultant surgeon. All operations were approached via a small postauricular incision, elevation of subperiosteal flaps through cortical mastoidectomy, posterior tympanotomy, and round window approach. Medel Sonata computerized cochlear implant device (Medel Medical Electronics, Innsbruck, Austria) was utilized to obtain the e-ECAPs. This device stimulates the auditory nerve in the cochlea to measure the response through the implant electrode array by an automatic auditory response telemetry (ART) software tool (AutoART), which automatically measures and analyzes all electrode channels.

In the AutoART, the stimulation intensity is continuously increased. This FineGrain method enables a very precise e-ECAP threshold estimation for each electrode. Once the e-ECAP threshold is determined, the next electrode is stimulated. Verifying FineGrain e-ECAP thresholds on all 12 electrodes only takes ∼90 seconds. Subsequently, a clearly defined e-ECAP threshold for each electrode (lead) can be obtained.

### 2.3. Study Protocol

After implanting and placing the external coil, the first e-ECAP thresholds were recorded for all 12 leads. If the first measurement was in the light anesthesia (61–80), then the anesthetist made the anesthesia deeper (40–60) and the second measurement will be obtained, and vice versa. The anesthetist changed the depth of anesthesia from deep to light and vice versa only by adjusting the doses of propofol. Randomly, in a consecutive alternative manner, 20 patients had light anesthesia at the beginning and 19 patients had deep anesthesia first. In most cases, the leads recorded the e-ECAPs. However, in a few patients, certain leads did not record the e-ECAPs. This was documented as the “recordability” and “nonrecordability” of each lead and was compared between both types of anesthesia. Both the surgeon and the audiologist were blinded to the anesthetic depth.

### 2.4. Statistical Analysis

Data were entered into a spreadsheet. Statistical analyses were performed using IBM SPSS Statistics Software (v.21), 2012. Data were presented as frequency distributions for categorical variables (sex and recordability of the leads) and mean ± standard error of the mean for continuous variables (age and e-EACP for all leads). Data were tested at a significance level of 0.05%. Independent sample *t-test* was applied to examine the significance level for the continuous variables with two levels of independent variable. This test was used to study the effect of both types of anesthesia “light versus deep” (independent or grouping variable) on the e-ECAP thresholds (dependent variable) for each lead. Also, it was used to compare the mean of e-ECAP thresholds (dependent variable) between males and females (independent or grouping variable). Moreover, a simple linear regression test was used to predict the relationship between the two continuous variables. It was used to correlate between the mean of e-ECAP thresholds (dependent variable) and age of patients (independent variable). Furthermore, Pearson's *χ*^2^ test was used to investigate the significance of association between categorical variables. It was used to investigate the relation between types of anesthesia and recordability of the e-ECAP. Finally, the absolute differences between the two thresholds for each lead (for whom the e-ECAP became lower versus became higher) from zero was studied to assess the individual differences. Also, individual scattered plots were provided.

## 3. Results

In this study, 39 patients were eligible for cochlear implant due to severe congenital sensory neural hearing loss and were enrolled during the study period. For all patients (pediatrics and adults), the hearing loss was congenital and sensory neural hearing loss. The study group included 18 males and 21 females. The mean age of the individuals was 8.1 years; the youngest one was 1 year old, and the oldest was 48 years old. Only 8 individuals were more than 17 years old. No statistically significant relationship was detected between the age and the amplitudes of the e-ECAPs for each lead in both deep and light anesthesia. In addition, no difference between males and females in the amplitudes of the e-ECAPs for each lead in both light and deep anesthesia.

**Figure 1:**
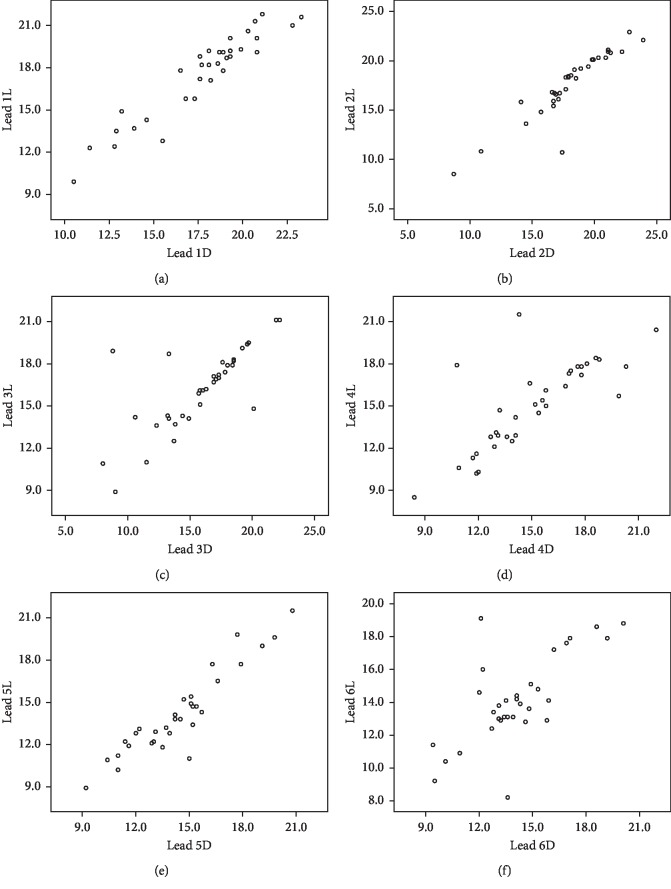
Scatter plots for the individual amplitudes of e-ECAP in the light and deep anesthesia in lead 1 (a), lead 2 (b), lead 3 (c), lead 4 (d), lead 5 (e), and lead 6 (f). The absolute differences showed no statistical difference. Thus, the variation is not clinically relevant.

**Figure 2:**
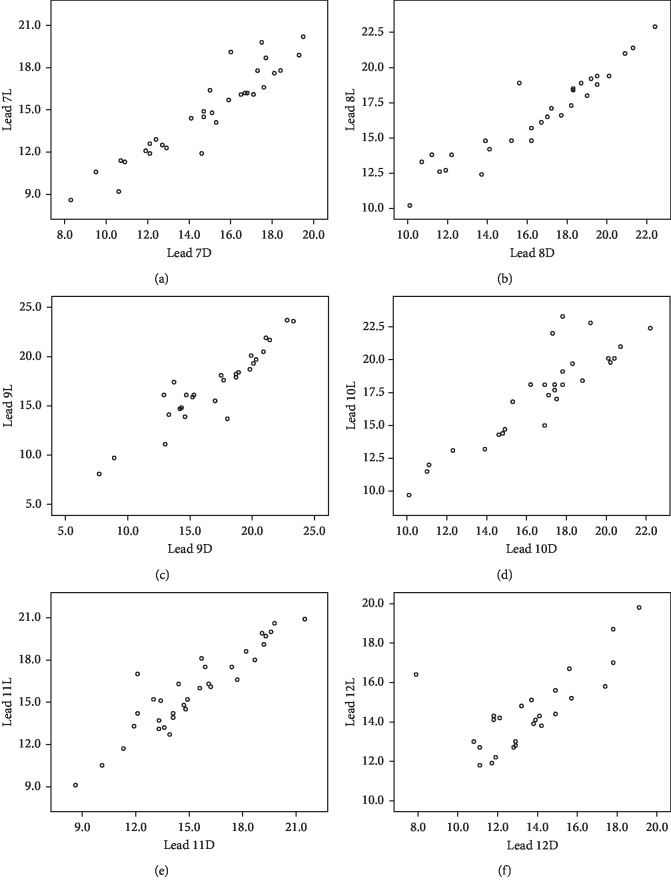
Scatter plots for the individual amplitudes of e-ECAP in the light and deep anesthesia in lead 7 (a), lead 8 (b), lead 9 (c), lead 10 (d), lead 11 (e), and lead 12 (f). The absolute differences showed no statistical difference. Thus, the variation is not clinically relevant.

After comparing the e-ECAP means for each electrode (lead) between the light and deep anesthesia, no significant differences were detected in each group.  Table 1 summarizes the mean values for each electrode. In addition, when comparing the absolute difference from zero in each lead for patients whom the e-ECAP thresholds became lower at a certain type of anesthesia and for patients whom the e-ECAP thresholds became higher at the same type of anesthesia, no statistical difference was detected. Accordingly, the nonsignificant difference between the light and deep anesthesia is a true relation, that is, a result from a nonclinically relevant individual variation in the same lead. Figures 1 and 2 show the scatter plots for the individuals in each lead. Also, the nonrecordable measurements showed no statistical difference between the light and deep anesthesia (Table 2).

## 4. Discussion

Cochlear implant devices are able to measure the e-ECAP thresholds from the auditory nerve. Each electrode directly stimulates the auditory nerve when electrical current necessary to trigger a hearing sensation passes. The amount of electrical current necessary to trigger the hearing sensation is different for each individual and for each stimulation canal [8]. Therefore, each user's speech processor must be individually adjusted together with each stimulation canal in a process that is called programming or mapping.

The e-ECAP thresholds obtained from the ART are routinely used to program the cochlear implant, especially in children, with the aim of predicting the appropriate limits for implant stimulation setting. Subsequently, this allows achieving an optimum dynamic range. This is a very important step for proper hearing and the successful use of cochlear implants [5, 6]. It is important to evaluate the effect of anesthesia on the intraoperatively acquired e-ECAP measurements.

Several studies proved that the influence of the TIVA gives more consistent responses than volatile anesthetics on electrically ESRT measurements [3, 8, 9]. Crawford et al. revealed that volatile anesthetics suppressed the stapedius reflex in a dose-dependent manner, whereas the e-ECAP was unaffected by the concentration of volatile anesthetic or propofol [3]. However, they did not study the effect of the TIVA on the e-ECAP thresholds. Also, Hejazi et al. suggested that during the cochlear implant surgery, the use of inhalational anesthetics should be avoided to achieve controlled pressure because this may suppress or even fully eliminate the stapedial reflex [10].

In addition, Stronks et al. concluded that isoflurane increases the threshold of e-ECAP in a dose-dependent manner [11]. A similar conclusion was drawn indirectly by another study where the e-ECAP thresholds were significantly lower when measured within the first 24 hours postoperatively than when measured intraoperatively [12]. It was suggested that neuronal sensitivity to electrical stimulation could be restored during this postoperative period. However, the possible effect of anesthesia was not considered [12]. This suggestion is strengthened by the results of Jana et al. study where they deduced that the intravenous anesthesia with nitrous oxide had no effect on determining the auditory thresholds in relation to the maximum comfort level values three months after implantation [8].

The mechanism underlying the change between the TIVA and inhalational anesthesia and between the mechanism of ESRT and ECAP is postulated. The neuronal arrangement of the stapedius reflex arc is polysynaptic and includes the auditory nerve as the afferent limb; central auditory brainstem connections involving the ventral cochlear nucleus, trapezoid body, and medial superior olive; and the efferent motoneurons that course with the facial nerve to the stapedius muscle [3]. Volatile anesthetics depress synaptic conduction more than axonal conduction [3]. Accordingly, oligosynaptic pathways are minimally affected, whereas polysynaptic pathways are exquisitely sensitive to the anesthetic concentration. Also, volatile anesthetics act at the neuromuscular junction by binding to protein sites within the nicotinic acetylcholine receptor and therefore channels blockade [3]. On the other hand, the ECAP thresholds are minimally affected by anesthesia because of the fact that these are axonal responses and suggest that the site of anesthetic action on the ESRT is unlikely to be the auditory nerve. Moreover, volatile anesthetics, unlike the intravenous anesthesia, appear to achieve their centrally depressive effects by the interference with neuronal membranes, which leads to a general depression of the central functions [9].

According to more recent studies on the depth of anesthesia, a significant influence on the e-ECAP thresholds was found and it was important to reduce the depth to achieve better results [5]. Moreover, a reduction in the depth of anesthesia may be helpful to obtain recordable results when e-ECAP cannot be recorded for some electrodes during the operation [5]. However, in that study, an inhalational anesthetic (isoflurane) was used and the effect of the depth of TIVA on the e-ECAP thresholds was not studied.

Also, the role of the TIVA was investigated in the spinal surgery. It has been shown that with the relative ease of TIVA administration, the need for inhalational anesthetics can be minimized. Various combinations of intravenous anesthetic regimens have been described and tested intraoperatively. Although propofol does demonstrate a dose-dependent reduction in the motor-evoked potential amplitude without effect on latency, it has repeatedly been shown to produce a more stable neurophysiological environment for monitoring, when compared with the inhalational anesthetics [13, 14].

Regarding the nonrecordable e-ECAP measurements of some leads during the study, many factors could explain the nonrecordability. Factors include three stimulation levels, intracochlear test electrode location, the separation between stimulating and recording electrodes, and stimulus polarity [15]. For example, an e-ECAP recorded at the apical electrodes tends to have higher amplitudes than those recorded at the basal electrodes [15]. In this study, the number of electrodes that did not record the e-ECAP was not different between the light and deep anesthesia.

This study is not without limitations. Firs, the data collection for the study was stopped due the change in the referral system. Second, we did not depend on the prestudy sample size calculation. This is because of the limited study period and the occasional performance of such types of surgeries. We aimed to collect all cases within the study period. Third, it is not proved completely that the BIS values have the same effect for pediatrics compared to adults; however, many authors stated no differences [16]. Fourth, we did not address the postoperative values of the e-ECAP, this is because that every patient in this study received the same anesthesia (deep and light). Fifthly, the e-ECAP was higher in light anesthesia in the number of patients and vice versa. This variance may be due to the individual effect on the e-ECAP; however, this variance is not clinically relevant as the absolute difference from zero between the two thresholds is not statistically significant. This would confirm that the depth of anesthesia may have minimal effect. Finally, the other parameters for e-ECAP as peak latency level were not collected.

## 5. Conclusion

To the best of our knowledge, this is the first study to investigate the effect of the depth of TIVA on the e-ECAP thresholds in cochlear implantation operations.

TIVA may be administered with no effect on the intraoperative reading of the e-ECAP thresholds, which could influence the adjustment of the electrodes and possible interference with the hearing ability or quality in the future. Larger studies and research would be recommended to establish the best protocol of anesthesia for cochlear implant patients.

## Figures and Tables

**Table 1:** The e-ECAP measurements in different leads within light and deep anesthesia.

	Light anesthesia, mean ± SE (*μ*V)	Deep anesthesia, mean ± SE (*μ*V)	*P* value^*∗*^
Lead 1	17.36 ± 0.48	17.72 ± 0.53	NS
Lead 2	17.55 ± 0.63	17.98 ± 0.55	NS
Lead 3	16.00 ± 0.50	15.47 ± 0.61	NS
Lead 4	14.98 ± 0.51	15.01 ± 0.49	NS
Lead 5	14.50 ± 0.57	14.48 ± 0.45	NS
Lead 6	14.27 ± 0.468	14.02 ± 0.472	NS
Lead 7	14.52 ± 0.51	14.59 ± 0.52	NS
Lead 8	17.19 ± 0.93	16.77 ± 0.79	NS
Lead 9	17.45 ± 0.74	17.29 ± 0.67	NS
Lead 10	17.80 ± 0.60	16.82 ± 0.53	NS
Lead 11	15.65 ± 0.496	15.27 ± 0.504	NS
Lead 12	14.10 ± 0.48	14.27 ± 0.91	NS

SE: standard error; NS: not significant; *μ*V: microvoltage. ^*∗*^Statistical test: independent sample *t*-test.

**Table 2:** Number of nonrecordable leads within light and deep anesthesia.

	Light anesthesia, *N* (*n*1), *N* = 39	Deep anesthesia, *N* (*n*2), *N* = 39	*P* value^*∗*^ (*P* value 2)
Lead 1	2 (0)	5 (3)	NS (NS)
Lead 2	5 (2)	5 (2)	NS (NS)
Lead 3	3 (2)	2 (1)	NS (NS)
Lead 4	3 (1)	2 (0)	NS (NS)
Lead 5	5 (3)	4 (2)	NS (NS)
Lead 6	5 (4)	3 (2)	NS (NS)
Lead 7	2 (2)	5 (5)	NS (NS)
Lead 8	6 (4)	6 (4)	NS (NS)
Lead 9	7 (4)	7 (4)	NS (NS)
Lead 10	5 (3)	8 (6)	NS (NS)
Lead 11	4 (2)	4 (2)	NS (NS)
Lead 12	11 (1)	12 (2)	NS (NS)

*N*: number of total nonrecordable cases; NS: not significant; *n*1: is the number of leads that have been changed in the deep anesthesia and became (or were) recordable; *n*2: number of leads that have been changed in the light anesthesia and became (or were) recordable; *P* value 2 is the *P* value for the change in the recordability. ^*∗*^Statistical test: Pearson's *χ*^2^ test.

## Data Availability

The datasets generated and analyzed during the current study are available from the corresponding author upon request.
